# Self-limiting fall armyworm: a new approach in development for sustainable crop protection and resistance management

**DOI:** 10.1186/s12896-022-00735-9

**Published:** 2022-01-27

**Authors:** Catherine E. Reavey, Adam S. Walker, Stephen P. Joyce, Lucy Broom, Alan Willse, Kyla Ercit, Mattia Poletto, Zoe H. Barnes, Thea Marubbi, Bartlomiej J. Troczka, David Treanor, Katherine Beadle, Ben Granville, Vanessa de Mello, Joss Teal, Edward Sulston, Anna Ashton, Luxziyah Akilan, Neil Naish, Oliver Stevens, Nerys Humphreys-Jones, Simon A. J. Warner, Sian A. M. Spinner, Nathan R. Rose, Graham Head, Neil I. Morrison, Kelly J. Matzen

**Affiliations:** 1 Oxitec Ltd, 71 Innovation Drive, Milton Park, Abingdon, OX14 4RQ UK; 2 Bayer Crop Science, Chesterfield, MO 63017 USA; 3 Present Address: Oxford University Innovation, Buxton Court, 3 West Way, Oxford, OX2 0JB UK; 4 Centre for Medicines Discovery, University of Oxford, Old Road Campus Research Build, Roosevelt Dr, Headington, Oxford, OX3 7DQ UK

**Keywords:** *Spodoptera frugiperda*, Fall armyworm, Resistance management, *Bt* crops

## Abstract

**Background:**

The fall armyworm, *Spodoptera frugiperda*, is a significant and widespread pest of maize, sorghum, rice, and other economically important crops. Successful management of this caterpillar pest has historically relied upon application of synthetic insecticides and through cultivation of genetically engineered crops expressing insecticidal proteins (*Bt* crops). Fall armyworm has, however, developed resistance to both synthetic insecticides and *Bt* crops, which risks undermining the benefits delivered by these important crop protection tools. Previous modelling and empirical studies have demonstrated that releases of insecticide- or *Bt*-susceptible insects genetically modified to express conditional female mortality can both dilute insecticide resistance and suppress pest populations.

**Results:**

Here, we describe the first germline transformation of the fall armyworm and the development of a genetically engineered male-selecting self-limiting strain, OX5382G, which exhibits complete female mortality in the absence of an additive in the larval diet. Laboratory experiments showed that males of this strain are competitive against wild-type males for copulations with wild-type females, and that the OX5382G self-limiting transgene declines rapidly to extinction in closed populations following the cessation of OX5382G male releases. Population models simulating the release of OX5382G males in tandem with *Bt* crops and non-*Bt* ‘refuge’ crops show that OX5382G releases can suppress fall armyworm populations and delay the spread of resistance to insecticidal proteins.

**Conclusions:**

This article describes the development of self-limiting fall armyworm designed to control this pest by suppressing pest populations, and population models that demonstrate its potential as a highly effective method of managing resistance to *Bt* crops in pest fall armyworm populations. Our results provide early promise for a potentially valuable future addition to integrated pest management strategies for fall armyworm and other pests for which resistance to existing crop protection measures results in damage to crops and impedes sustainable agriculture.

**Supplementary Information:**

The online version contains supplementary material available at 10.1186/s12896-022-00735-9.

## Background

The fall armyworm, *Spodoptera frugiperda*, is a significant and widespread pest of maize, sorghum, rice, and other economically important crops [1], and is now a significant global threat to agricultural productivity and food security. This moth is native to parts of the New World, is widespread across both North and South America, and is one of the most serious pests of maize in the Americas [2]. The caterpillars feed on crop plants, resulting in yield reductions and management costs valued at an estimated US$1 billion annually in Brazil alone [3–6]. In 2016, the fall armyworm was detected in sub-Saharan Africa for the first time, and has since spread rapidly across Africa, India, southeastern Asia and Australia [7–10]. The insect is equally destructive in its invasive range, causing an estimated one-third reduction in overall maize yield in some countries, costing farmers an estimated US$2.5–$6.3 billion in yield losses annually [11, 12].

Management of the fall armyworm has historically relied heavily on application of synthetic insecticides [13], but the often cryptic feeding behavior of larvae and, increasingly, resistance to a broad range of chemistries, can limit their effectiveness [2, 14]. Since 1996, corn varieties engineered to express insecticidal proteins from the bacterium *Bacillus thuringiensis* (*Bt*) have been available to growers, and are now planted on over 100 million acres globally [15–17]. In addition to providing protection to farmers’ crops, the widespread adoption of *Bt* crops has led to a global reduction in insecticide applications of 50 million kg on maize alone between 1996 and 2011 [18]. This, in addition to the taxonomic specificity of *Bt* toxins relative to synthetic insecticides and the fact that only those pests feeding on crops are affected, has led to reduced negative impacts on non-target species and helps to maintain biological control services provided by parasitoids and predators [15, 19].

To delay the development of pest resistance to these biotech crops, a high-dose/refuge strategy is typically recommended: pest insects feeding on a *Bt* crop receive a high dose of the insecticidal protein, and non-*Bt* ‘refuge’ crops are planted to allow a reservoir of *Bt*-susceptible insects to survive [20]. Due to challenges with implementation, wild populations of fall armyworm have developed resistance to many of these insecticidal proteins in biotech crops [14, 21–24].

A novel and complementary resistance management approach involves releases of insecticide-susceptible insects [25]. Such releases provide a source of insects carrying insecticide susceptibility alleles in addition to those arising from refuge plants, and so may act as a potent tool to protect the efficacy of insecticides and *Bt* crops. Conducting male-only releases is highly preferable, to minimize assortative mating between co-released susceptible insects and to avoid increases in eggs laid on the crop, but manual sex-sorting of insects is often inefficient and costly at scale [26]. This challenge has been overcome through the development of genetically engineered, male-selecting, self-limiting strains of pest insects, including in two lepidopteran pests: the pink bollworm (*Pectinophora gossypiella*) and the diamondback moth (*Plutella xylostella*) [27]. In these strains, sex-alternate splicing components of a sex determination gene—*doublesex* (*dsx*) in Lepidoptera—are coupled to the ‘tetracycline off’ (tet-off) system [27–29], resulting in upregulation of the tetracycline-repressive transcriptional activator (tTAV) in females only. Accumulation of tTAV in females occurs via a positive feedback loop with a tetracycline-responsive operator sequence [27, 30, 31] and induces female-specific mortality in pre-adult stages in the absence of dietary tetracyclines. Large male-only cohorts can thus be easily produced and released into the field [32]. When these self-limiting males mate with wild females, their offspring will inherit a copy of the self-limiting gene, resulting in mortality of female offspring: with sustained releases the target pest population is thereby suppressed. In addition, provided the self-limiting strain is developed using an insecticide-susceptible genetic background, the survival of the male offspring of self-limiting males and wild females will lead to introgression of insecticide-susceptibility alleles from the self-limiting strain into the target pest population [33]. Population modelling and glasshouse studies have shown that releases of males carrying a male-selecting trait can achieve both population suppression and dilution of *Bt* resistance alleles in the target population [25, 34, 35]. The species-specific effect of this strategy, coupled with a lack of toxicity of introduced proteins [36, 37], is anticipated to provide pest management with a low ecological impact. The introduced female-specific trait is also self-limiting in the absence of a dietary antidote, declining to extinction in the generations after releases stop [38]. Applied to fall armyworm, this self-limiting approach has the potential to address the challenge of resistance to *Bt* maize, to support management of this pest and protect the value of these *Bt* maize varieties to farmers.

Here, we describe the first reported germline transformation of fall armyworm and the development of a male-selecting, self-limiting strain, called OX5382G. To investigate the potential of OX5382G as a viable future crop protection tool, we tested the mating competitiveness of adult males, confirmed the self-limiting nature of the OX5382G transgene in laboratory populations, and modelled the effect of releasing male-selecting, self-limiting OX5382G adult moths into target fall armyworm populations.

## Results

### Characterisation of fall armyworm *doublesex *gene (*Sfdsx*)

**Fig. 1:**
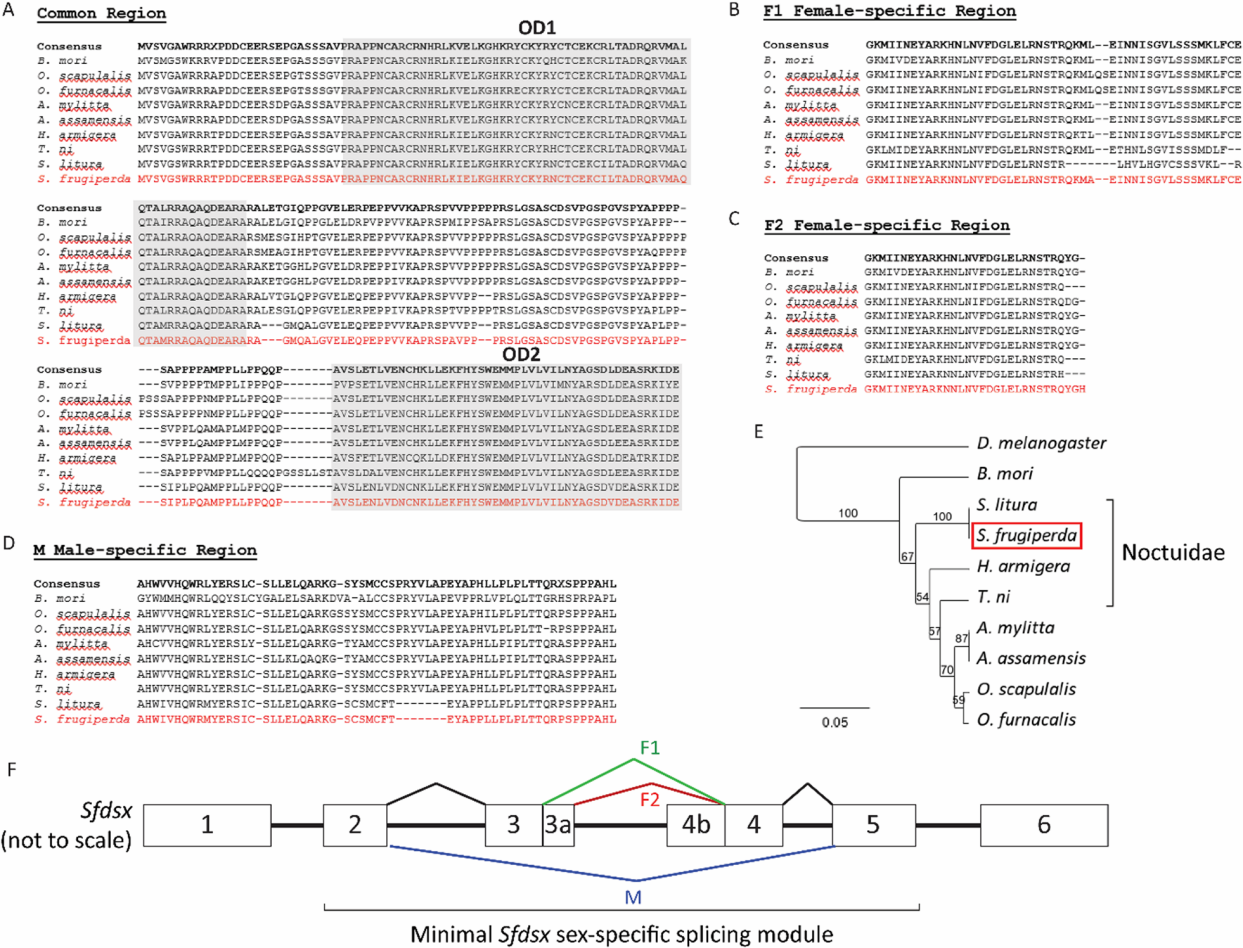
Phylogenetic analyses of DSX homologues. **A**–**D** Multiple sequence alignment of DSX protein isoforms in Lepidoptera. The sequences are divided into common male and female region (**A**), F1 and F2 female-specific region (**B** and **C**, respectively) and male-specific region (**D**). The DNA binding (OD1) and oligomerisation (OD2) domains are boxed and *S. frugiperda* DSX protein is highlighted in red. **E.** Phylogenetic tree based on the combined OD1 and OD2 DSX protein sequences in Lepidoptera and *D. melanogaster.* Bootstrap support is indicated above each branch. **F.** Schematic representation of *Sfdsx* gene splicing. The minimal sex-specific splicing module, spanning exons 2 to 5 is highlighted. Differential splicing is indicated above (F1 and F2 isoforms) and below (M splice form) the gene

To engineer female-specific expression of tTAV in fall armyworm, we used sequences from the endogenous *dsx* gene in this moth. Protein sequences shared between the male and the female *dsx* transcripts in two Lepidoptera—the silkworm, *Bombyx mori* (*Bmdsx*) and the corn earworm, *Helicoverpa armigera* (*Hadsx*)—were used to identify putative *Sfdsx* regions from the ASM75363v2 *S. frugiperda* draft genome assembly [39]. *Bmdsx* is well-characterised and the gene has been shown to undergo alternative splicing to generate at least one male-specific splice form (*Bmdsx M*) and at least two female-specific splice forms (*Bmdsx F1* and *Bmdsx F2*) [40, 41]. In addition to these, several newly characterised sex-specific splice forms may exist [42]. *Hadsx* has also been shown to splice in a similar manner [43]. Unbiased structural modelling of the best match aligned well with the *Drosophila melanogaster* structure of *dsx* long isoform (entry 2JZ1) deposited at the RCSB protein data bank, strongly suggesting that the sequence identified belonged to *Sfdsx.* Further hits were obtained from the SPODOBASE EST database [44] and from the transcriptome assembly obtained from *Sf*21 cells [45]. Predicted translation of the scaffolds obtained from the *Sf*21 transcriptome dataset generated a high-quality alignment with DSX proteins from other lepidopteran species (Fig. 1). Furthermore, phylogenetic analyses also placed the predicted *S. frugiperda* protein within the Noctuidae family (Fig. 1). Finally, publication of the latest chromosome-level assembly (ZJU_Sfru_1.0) for fall armyworm allowed us to confirm that *Sfdsx* is located on chromosome 21. Primers specific to the predicted exonic regions of *Sfdsx* were designed and the susceptible *S. frugiperda* strain held by Oxitec [46, 47] was sequenced across the region spanning exons 2 and 5, which constitutes the minimal sex-specific splicing module of *Sfdsx* (Fig. 1).

### Construct design for self-limiting and marker phenotypes

**Fig. 2:**
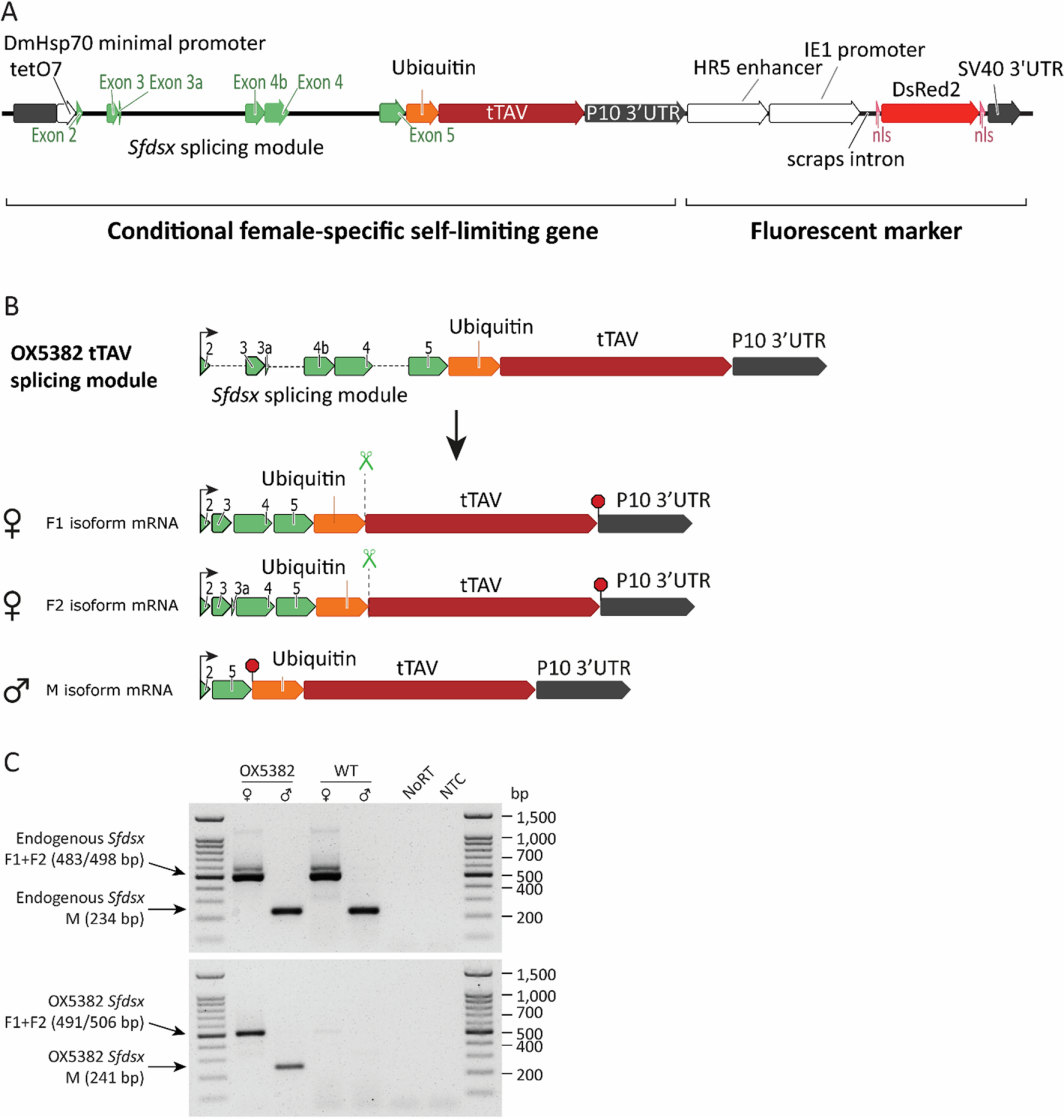
OX5382 construct schematics and splicing. **A** Linear construct map for OX5382. The position of the conditional female-specific self-limiting gene and that of the fluorescent marker is indicated. **B** Predicted splicing pattern of OX5382. The mRNA is predicted to generate three major sex-specific splice forms: F1, F2 and M. The F1 and F2 female-specific splice forms are in-frame with tTAV, while the male-specific M isoform is not. Translation start sites are indicated by an arrow; in-frame stop codons are indicated by red octagons. The ubiquitin cleavage site in the translated F1 and F2 DSX proteins is indicated by scissors. **C** Representative mRNA splicing analysis on OX5382 G_1_ adults reared on tetracycline. Both endogenous *Sfdsx* (top gel) and construct-derived OX5382 *Sfdsx* show the predicted sex-specific splicing pattern. Non-injected wild-type individuals were included in the assay as a negative control for the construct-specific splicing PCR. NoRT: non-reverse transcribed control; NTC: no template control

A synthetic splicing module was created by using exonic fragments from the sequenced *Sfdsx* gene, as well as 5′ and 3′ fragments of the intervening introns. The splicing module was coupled to a tet-off system by linking the synthetic splicing cassette to a sequence encoding tTAV and to a tetracycline-responsive operator sequence (*tetO*_7_) [27–29] (Fig. 2). The open reading frame of the *Sfdsx* gene was modified in pOX5382 so that the *Sfdsx F1* and *Sfdsx F2* female-specific splice forms could be translated in-frame with tTAV in female individuals. The sequence was modified in such a way that the *Sfdsx M* male-specific splice form encodes a stop codon at the end of *Sfdsx* exon 5, and therefore male individuals were not expected to produce any tTAV protein (Additional file 1: Supplementary Figure 1)(Fig. 2). The synthetic *Sfdsx_tTAV* gene in pOX5382 is regulated by a minimal promoter and 5′ UTR from the *D. melanogaster* heat shock protein 70 (*DmHsp70*) gene (Fig. 2). Additional gene expression control is provided by a P10 3’UTR, derived from the *Autographa californica* multicapsid NPV (AcMNPV) baculovirus [48]. The ubiquitin protein coding sequence was placed between the *Sfdsx* and tTAV sequences. Ubiquitin is cleaved through normal cellular processes, so that the *Sfdsx*-derived amino acids and those from ubiquitin are removed, producing unmodified tTAV [49].

To permit visual discrimination of transgenic individuals from wild-type counterparts, a fluorescent protein marker was added to the transformation constructs. This consisted of a DsRed2 protein sequence, expression of which is regulated by a hr5-*ie1* enhancer-promoter derived from AcMNPV.

These construct components were assembled, flanked by terminal sequences from the *piggyBac* transposable element, to form the pOX5382 construct.

### Development of a male-selecting, self-limiting strain of fall armyworm

**Fig. 3:**
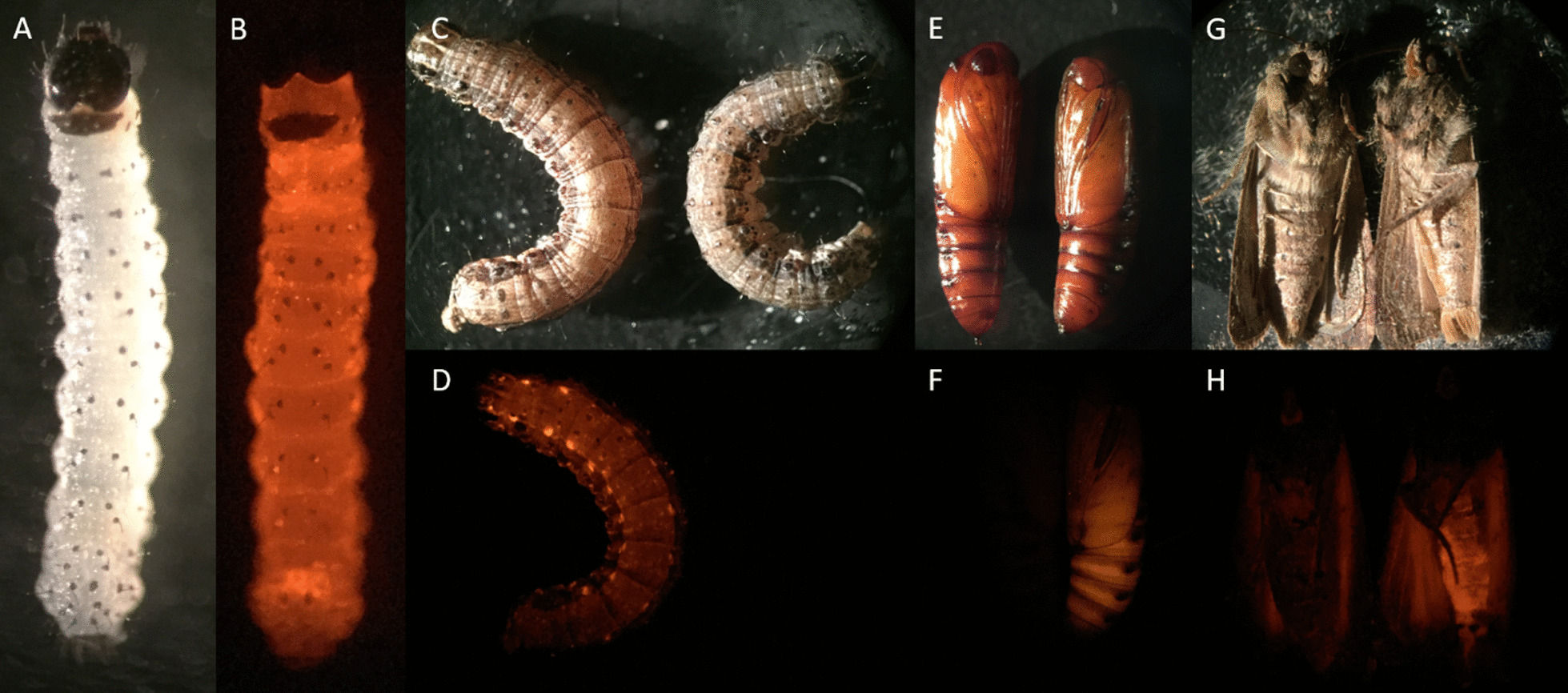
The DsRed2 fluorescent protein marker is clearly visible in OX5382G insects across all post-egg life stages under appropriate filters, making them distinguishable from wild-type counterparts. Panels show (**A)** OX5382G first-instar larva under white light and (**B)** and under DsRed2 filters; (**C)** OX5382G (left) and wild-type (right) later-instar larvae under white light and (**D)** and under a DsRed2 filters;** E** wild-type (left) and OX5382G (right) pupae under white light and (**F**) under DsRed2 filters; and (**G**) wild-type (left) and OX5382G (right) adults under white light and (**H**) under DsRed2 filters

A total of 45,133 fall armyworm embryos were injected with pOX5382 and a transposase source, resulting in 3,808 G_0_ survivors. Screening G_1_ larval progeny for the presence of expressed DsRed2 enabled identification of 12 independent transformation events (0.3% transformation rate). Five of these OX5382-transformed strains—OX5382A, OX5382B, OX5382C, OX5382G and OX5382J—were successfully established in culture. The DsRed2 marker was visible in all post-egg life stages when viewed under the fluorescence microscope. The synthetic OX5382 *Sfdsx* cassette was confirmed to show the predicted splicing pattern in transformed individuals, with males expressing only the *Sfdsx M* isoform and females showing expression of *Sfdsx F1* and *Sfdsx F2* (Fig. 2)*.* The brightness of fluorescence varied between OX5382 strains and life stages but in strain OX5382G, for example, DsRed2 was clearly visible, allowing DsRed2-expressing insects to be easily distinguished from wild-type counterparts (Fig. 3).

**Fig. 4:**
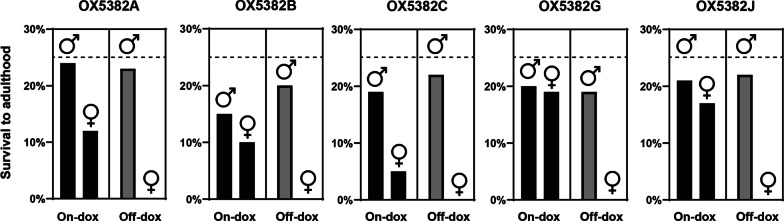
OX5382G male and female survival to adulthood when reared on larval diet that either contained doxycycline or was doxycycline-free (on-dox and off-dox, respectively). Percentages of OX5382G-hemizygous first-instar larvae reaching adulthood are shown for transgenic males and females reared on- and off-dox. Larvae used in these assays were the progeny of crosses between OX5382-hemizygous and wild-type adults; as such, following Mendelian inheritance and assuming equal survival rates across phenotypes, a 1:1:1:1 ratio of OX5382-hemizygous male, OX5382-hemizygous female, wild-type male, and wild-type female larvae is expected, and so full survival of one of these categories would be approximately 25% of all survivors in that cohort, as indicated by a dashed line on the charts. Data is only shown for the transgenic progeny of these crosses

Two of the OX5382 strains—OX5382G and OX5382J—displayed the designed female-specific mortality when doxycycline (the selected tetracycline analogue) was withheld from the larval diet, and high levels of OX5382-hemizygous female survival (close to the expected 25% of total progeny) when doxycycline was included in larval diet (Fig. 4). In both strains, larval mortality in the absence of doxycycline occurred during the earlier stages of larval development. With OX5382J, scoring progeny phenotypes from back-crosses and PCR genotyping indicated low survival rates of OX5382J-homozygous females. Therefore, of the 12 transgenic strains created using the pOX5382 construct, only OX5382G was taken forward to establish a homozygous colony from 117 founding individuals.

### Mating competitiveness of OX5382G adult males

Mating competitiveness tests were performed under laboratory conditions to assess whether OX5382G males compete successfully for females with wild-type males. OX5382G-homozygous adult males, reared in the absence of doxycycline, achieved 44.1% of copulations, showing no significant difference in mating success relative to wild-type male competitors (generalized linear model *t* = -1.23, *p* = 0.22). The proportion of copulations achieved were measured in 13 replicate cages, and the variation among cages showed no evidence of overdispersion. OX5382G male pupae were slightly but significantly smaller (mean = 0.257 g) than wild-type male pupae (mean = 0.271 g) (generalized linear model *F*_1,198_ = 21.32, *p* < 0.001), but this did not appear to affect the mating competitiveness of OX5382G males.

### Post-release decline of OX5382G allele frequency

**Fig. 5:**
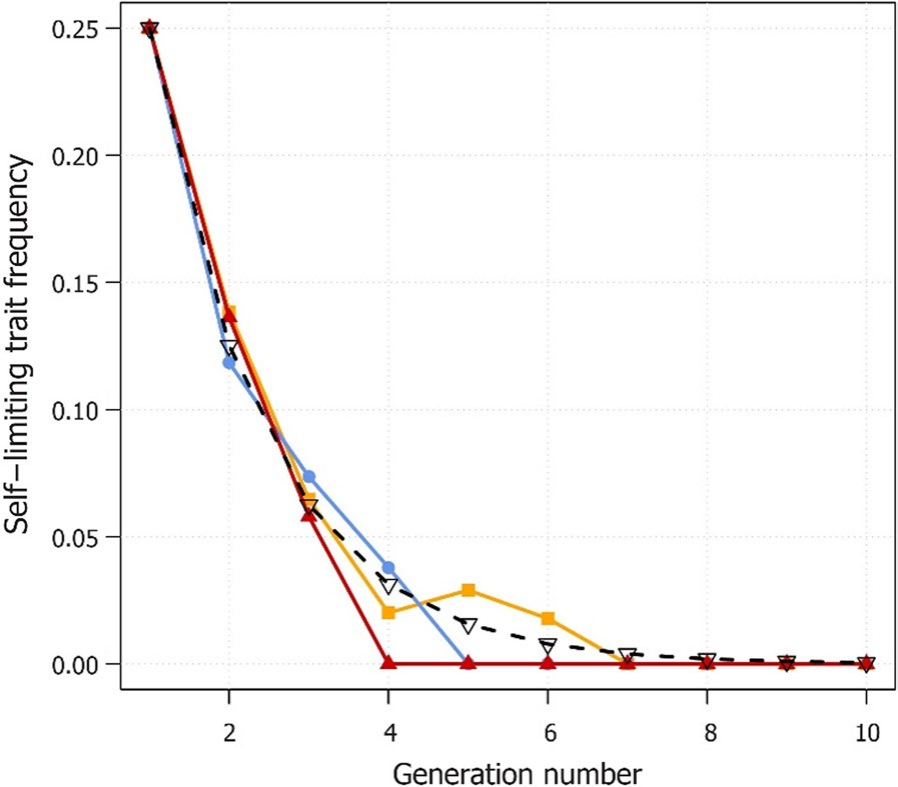
Decline of OX5382G allele frequencies in caged populations. Solid lines show frequency of the OX5382G allele in the three laboratory populations over successive generations. The dotted black line represents the expected values if the trait frequency halves every generation

To demonstrate anticipated decline of the male-selecting trait after field releases stop, a laboratory experiment was conducted to measure the rate at which the self-limiting OX5382G trait would decline to extinction in three replicated, otherwise wild-type populations (Fig. 5). In these three populations, OX5382G had fallen to extinction within 4, 5 and 7 generations after OX5382G releases had stopped (Fig. 5).

### Modelling the impact of OX5382G releases on pest populations

To help predict the impact of OX5382G fall armyworm releases over *Bt* crops, we modelled the impact of releases on the wild fall armyworm population size, and on the resistance allele frequencies for resistance to one or two plant-expressed *Bt* proteins, taking into account the over-flooding rates of OX5382G fall armyworm and the proportion of refuge planting.

**Fig. 6:**
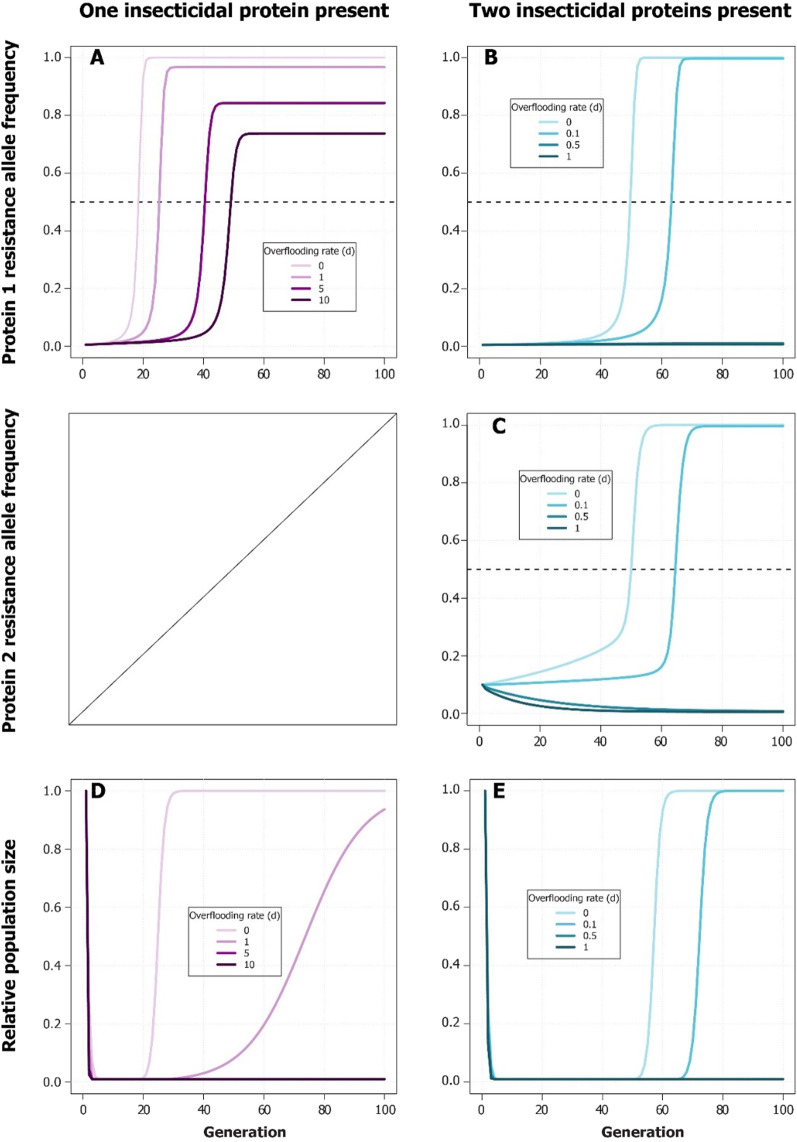
The over-flooding rates (the number of OX5382G male moths per wild male fall armyworm moth) of OX5382G necessary in computer simulations to delay the accumulation of insecticidal protein (*Bt*) resistance alleles (**A**, **B**, and **C**) and to suppress fall armyworm population (**D** and **E**) when either one or two *Bt*-producing genes are present in the host corn, and 10% of the field are non-*Bt* refuge plants. In plots **A**, **B**, and **C**, the horizontal dashed line marks the point at which the resistance allele frequency surpasses 0.5. In plot** D**, the ‘5’ over-flooding rate line cannot be seen because it is nearly colinear with the ‘10’ over-flooding rate line, and in plot** B** and** E**, the ‘0.5’ over-flooding rate line cannot be seen because it is nearly colinear with the ‘1’ over-flooding rate line

In modelled populations of fall armyworm, releases of self-limiting OX5382G fall armyworm males slowed or prevented the spread of resistance to insecticidal proteins produced by *Bt* corn, even with a low over-flooding rate (ratio of released OX5382G male moths to wild fall armyworm male moths) (Fig. 6). When refuge was held constant at 10% and female reproductive rate (*R*) was held at 5, in crops expressing a single insecticidal protein, a 10:1 over-flooding rate delayed the generation at which the resistance allele frequency (RAF) exceeded 0.5 from generation 19 to generation 50. Crops expressing two insecticidal proteins—a typical use case by commercial farmers—required even lower over-flooding rates to delay resistance than crops expressing one protein. At an over-flooding rate of 0.5:1, both Protein 1 and 2 RAF did not exceed 0.5 within the 100 generations modelled, whereas, when releases were absent, Protein 1 RAF exceeded 0.5 at generation [50] and Protein 2 at generation 51. (Fig. 6). When looking at changes in population size, the modelled populations initially declined sharply, but as *Bt* resistance alleles accumulate through selection, population size returns to carrying capacity in the absence of OX5382G releases. The initial crash in population size is almost entirely due to the *Bt* treatment, as the slope of the line (within the first five generations) in simulations where OX5382G releases were absent is nearly collinear to simulations where releases were added. When no OX5382G fall armyworm were released, population size returned to 99% of carrying capacity at generation 30 in the one-*Bt*-protein model, and generation [63] in the two-*Bt*-protein model. In the one-*Bt*-protein model, a 5:1 over-flooding rate was sufficient to keep population size from increasing again after the initial crash, and in the two-*Bt*-protein model, an over-flooding rate of 0.5:1 was sufficient (Fig. 6).

**Fig. 7:**
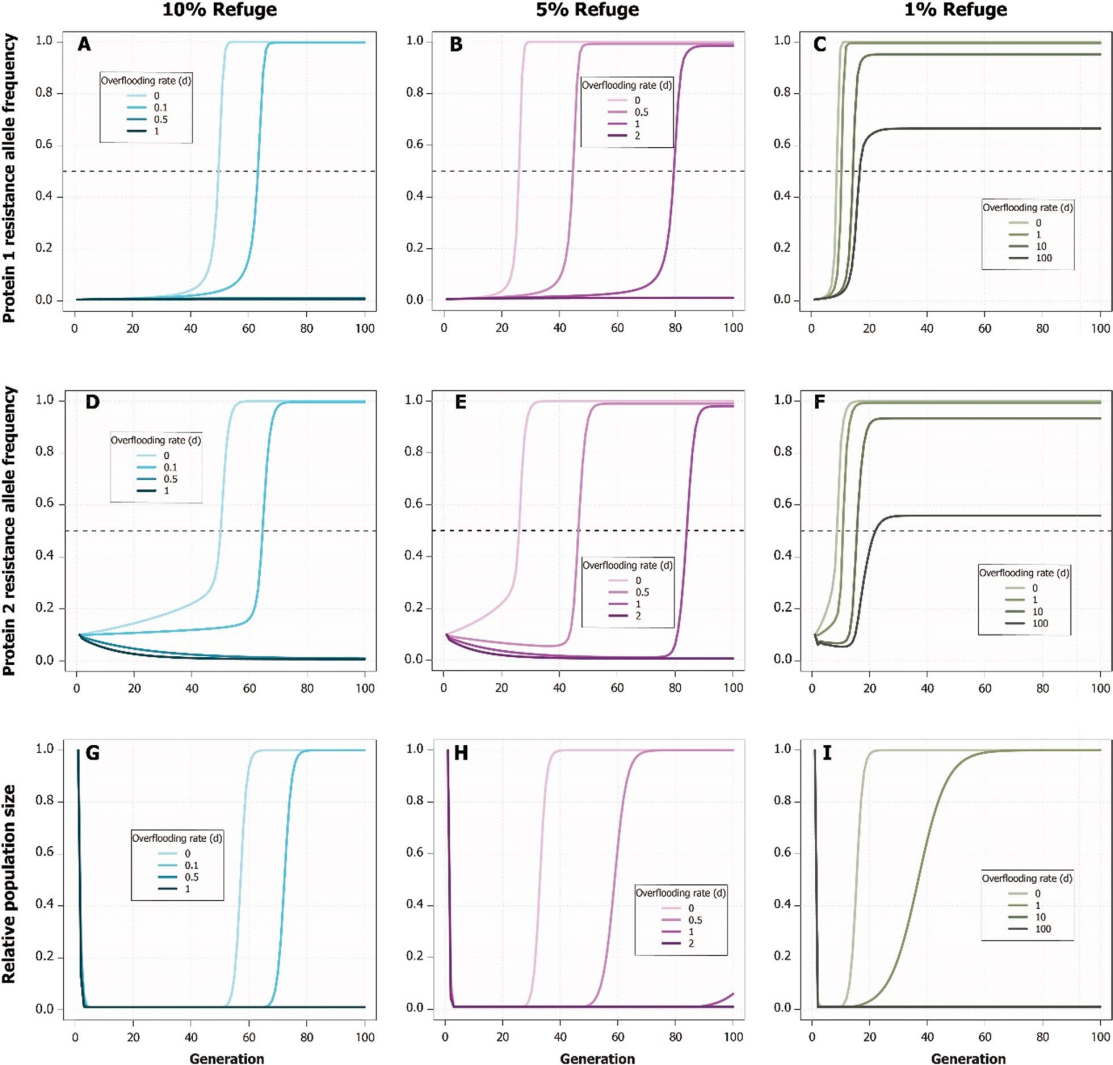
The effect of proportion of refuge plants on over-flooding rate (the number of OX5382G male moths per wild fall armyworm male moth) required to reduce resistance allele frequencies and suppress population in simulated populations of fall armyworm. In these simulations, two insecticidal proteins were present in the *Bt* crop. **A**, **B**, and **C** show the response in the frequency of the allele for protein 1 resistance, and **D**, **E**, and **F** show protein 2 resistance. **G**, **H**, and **I** show relative population size. In plots **A** to **F**, the horizontal dashed line marks the point at which the resistance allele frequency surpasses 0.5. In plot A and G, the ‘0.5’ over-flooding rate line cannot be seen because it is nearly collinear with the ‘1’ over-flooding rate line. In plot I, the ’10’ over-flooding rate line cannot be seen because it is collinear with the ‘100’ over-flooding rate line

Simulations showed that decreasing the proportion of refuge plants in a field had a detrimental effect on maintaining susceptibility to insecticidal proteins and on long-term population suppression of fall armyworm, but releasing OX5382G can lead to effective resistance reversal and compensate for inadequate refuge (Fig. 7). When 10% refuge was present, a 0.5:1 over-flooding rate was sufficient to prevent RAF for Proteins 1 and 2 from exceeding 0.5 and prevent population size from increasing again. When refuge was reduced to 5%, RAF increased more quickly in the absence of OX5382G releases, exceeding 0.5 by generation 26 for Protein 1 and 27 for Protein 2. In this case, a slightly higher over-flooding rate of 2:1 was necessary to prevent the fixation of Protein 1 and Protein 2 resistance and keep populations suppressed. When the proportion of refuge plants was reduced to 1%, the resistance allele frequency increased very quickly (in Protein 1, from 0.005 to over 0.5 in only nine generations), and the pest population began to grow again after eleven generations, in the absence of OX5382G releases. With such a low proportion of refuge, a higher fall armyworm over-flooding rate slowed, but did not prevent, the accumulation of resistance alleles. However, the simulated release of OX5382G insects in a field with 1% refuge did result in sustained population suppression at an over-flooding rate of 10:1 (Fig. 7).

## Discussion

Here, we described the first reported germline transformation of fall armyworm and the development of a male-selecting, self-limiting strain, called OX5382G (however, while this paper was being revised in response to reviewers’ comments, another report of germline transformation of *Spodoptera frugiperda* was also published [50]). The successful development of a male-selecting, self-limiting strain of the fall armyworm represents a promising future addition to the tools available to manage this highly damaging and invasive crop pest. Mating-based approaches to the suppression and eradication of agricultural pests, including Lepidoptera, have been in use for over 60 years [51] and genetically engineered strains of pests and disease vectors have been successful in significantly reducing target pest populations and have shown huge potential to improve the effectiveness and scalability of such approaches [52–54]. The development of a strain of fall armyworm engineered to show conditional, female-specific mortality offers a new pest management option and resistance management strategy where fall armyworm threatens the effectiveness of insecticides and *Bt* crops [25, 33, 35].

The OX5382G strain will enable genetic sexing for cost-efficient and large-scale production of male-only cohorts to be released over crops, thereby avoiding release of egg-laying female moths and mating between co-released moths. Mating competition assays described here demonstrate that OX5382G males are competitive against wild-type males, indicating that the OX5382G transgene will not impede the ability of OX5382G males to mate with wild females in the field. Our results also demonstrate that the female-specific mortality conferred by the OX5382G transgene restricts persistence of the transgene in fall armyworm populations: the gene is self-limiting, declining to extinction within a few generations. In future applications on crops, the OX5382G trait is therefore not expected to persist in fall armyworm populations after releases stop.

We explored the potential for releases of OX5382G males to delay the spread of insecticide resistance and suppress fall armyworm populations by simulating releases of OX5382G on *Bt* crops. Our simulations showed that without the release of OX5382G males, genetic resistance to insecticidal proteins increases rapidly due to natural selection. The presence of insecticidal proteins in biotech crops suppresses fall armyworm populations initially, but as the resistance allele frequency increases, population size rebounds and returns to carrying capacity. To mitigate this threat, our simulations show that releases of OX5382G moths, even in relatively small numbers, have the potential to delay the accumulation of resistance alleles in a fall armyworm population, and suppress the size of that population. These findings are consistent with those of previous modelling studies [25, 33] and empirical studies with another lepidopteran [35].

Incorporating a second insecticidal trait in the cultivated crop delays the accumulation of resistance alleles in the modelled population, even when the secondary protein is less effective and faces higher initial resistance in the fall armyworm population. Moreover, our simulations demonstrate that, relative to releases on a single-*Bt* protein crop, fewer self-limiting insects are required to reduce resistance rates and suppress the population when the crop produces two *Bt* proteins. In the modelled scenarios, resistance and pest management is most effective when the proportion of refuge is high (10%), when two insecticidal proteins are present in the planted crop, and when over-flooding rates are at least 0.5 OX5382G moth for every wild-type fall armyworm moth. With non-*Bt* refuge reduced to 5% of the crop, it was still possible to achieve population suppression and delay resistance if OX5382G were released. However, when refuge is further lowered to 1%, releasing even relatively large numbers of self-limiting fall armyworm did not prevent the accumulation of resistance alleles. Suppression of the target pest population is still induced by the addition of OX5382G in this low-refuge scenario, but it is likely that if releases were stopped, population size would quickly rebound because of the high frequency of resistance alleles in the remaining insects.

## Conclusions

The fall armyworm is well-suited to future management by release of self-limiting males: it reproduces sexually, it can be reared under artificial conditions, it is now difficult to control by conventional methods, and presents a significant problem when resistant to *Bt* crops. Further development work is under way to enable efficient and large-scale insect production and application. Understanding of the field performance of OX5382G males in dispersing, finding and mating with pest females, and persisting on crops, alongside population modelling incorporating these learnings as well as other factors, such as immigration and insect fitness costs associated with—for example—*Bt* resistance, will inform how future application on farmers’ crops will provide maximum benefit. Our population modelling results indicate that, of the potential uses that self-limiting fall armyworm could provide in the future, it shows particular promise as a tool to address the *Bt*-resistant fall armyworm challenge faced by farmers in the Americas; but this approach could also play a part in mitigating the more recent devastation of crops in Sub-Saharan Africa and beyond. Delaying development of resistance to effective *Bt* proteins may lead to more sustainable food production, with lower reliance on broad-spectrum sprayed insecticides. This anticipated benefit, together with the target-specificity and non-toxicity of self-limiting insects, would enable food production with a lower environmental impact.

Our results provide promise for a new and valuable addition to future integrated pest management programs for fall armyworm, and for other pests in which insecticide resistance has become a significant challenge for farmers. Preservation of, and reducing over-reliance on, existing tools whilst minimizing their ecological impact will improve food security, farmers’ livelihoods, and environmental sustainability.

## Methods

### Bioinformatics

Bioinformatic analyses were carried out using Geneious® Prime v2020.1.2. Sequence alignments were performed using the Geneious global alignment tool and the following protein sequences: *Bombyx mori* (XP_012544218.1, XP_012544234.1, XP_012544211.1 and XP_012544206.1), *Antheraea assamensis* (ADL40846.1, ADL40847.1, ADL40848.1, ADL40850.1 and ADL40852.1), *Antheraea mylitta* (ADL40853.1, ADL40854.1 and ADL40855.1), *Ostrinia furnacalis* (AHF81635.1, AHF81636.1, AHF81637.1, AHF81638.1, AHF81640.1 and AHF81646.1), *Ostrinia scapulalis* (BAJ25850.1, BAJ25851.1 and BAJ25852.1), *Trichoplusia ni* (XP_026734616.1, XP_026734782.1 and XP_026746964.1), *Spodoptera litura* (XP_022817435.1 and XM_022961667.1—translated) and *Helicoverpa armigera* (XP_021192052.1, XP_021192053.1, XP_021192054.1 and AHF81649.1). Phylogenetic analysis was performed using the combined DSX protein sequences spanning the OD1 and OD2 domains and using the DSX protein sequence from *D. melanogaster* (NP_731197.1) as an outgroup. The reliability of the dendrogram was tested by the bootstrap method [55]. Structural modelling was carried out using Phyre2 [56].

### Insect rearing

All strains were reared under standard insectary conditions: 25 °C [± 2 °C], 50% [± 10%] relative humidity, 12 h: 12 h light: dark cycle. Larvae were reared on commercially available beet armyworm diet (Frontier Scientific Services) in cellular rearing trays. For on-doxycycline rearing, doxycycline hydrochloride (Apollo Scientific) was added to the larval diet to a final concentration of 100 µg/ml; for off-doxycycline rearing, doxycycline was substituted with streptomycin sulphate. To prevent the cannibalistic behaviour typical in the larvae of this species, a single first-instar larva was introduced into each cell. Pupae were removed from rearing trays and allowed to eclose in plastic mesh cages, and the resulting adult moths were provided with 7.5% (w/v) sucrose solution (supplemented with 100 µg/ml doxycycline as required). Filter paper was added to cages as an ovipositional substrate, and eggs were allowed to hatch in Petri dishes containing a thin layer of larval diet. All eggs used for microinjections and adults used for crosses were from a non-modified wild-type strain, deriving originally from a colony of fall armyworm collected on corn from the US Department of Agriculture in Starkville, Mississippi (USA) in 2007, and subsequently infused with field-collected insects from the Union City area, Tennessee (USA). The strain has been maintained continuously in Oxitec’s UK laboratories since 2015, and is susceptible to commonly applied synthetic insecticides and *Bt* proteins [46, 47].

### Cloning and construct assembly

Constructs used in this study are non-autonomous *piggyBac* elements, which incorporate terminal sequences of the *piggyBac* transposable element. Only the DNA segment flanked by these sequences integrates in the insect genome. Transposition occurs only in the presence of exogenous *piggyBac* transposase, which is provided separately (‘helper’ mRNA) [57]. The *Sfdsx* splicing cassette was constructed by gene synthesis (ThermoFisher Scientific). Individual components were assembled by standard molecular biology techniques using the NEBuilder® HiFi DNA Assembly Cloning Kit (New England Biolabs). The plasmid backbone containing the pOX5382 constructs is based on cloning vector pKC26-FB2 (Genbank #HQ998855).

### Microinjection procedures and strain development

Fall armyworm eggs of the ‘Starkville’ wild-type background were micro-injected by standard methods [58, 59], injecting a combination of pOX5382 plasmid DNA (final concentration 500 ng/µl) and *piggyBac* mRNA helper (final concentration 350 ng/µl) as the source of transposase. The plasmid DNA and the transposase mRNA were reconstituted in an injection buffer (5 mM KCl, 0.1 mM NaH_2_PO_4_, pH 6.8) [57].

Adult injection survivors (generation 0 or G_0_) were placed in mating cages (30 × 30 × 30 cm). Each cage was populated with approximately 40 injection survivors. G_1_ first-instar larvae were hatched on beet armyworm diet containing activated charcoal to aid visual identification and were screened for DsRed2 fluorescence using a Leica, MZ10F microscope equipped with filters for DsRed2 detection: maximum excitation 563 nm, emission 582 nm. Transformed G_1_ insects were reared to adulthood and crossed to wild-type counterparts to be maintained as hemizygous strains.

### RNA splicing assays

Assays to assess RNA splicing of endogenous and construct-derived *dsx* were conducted on injected G_0_ or G_1_ larvae. RNA extraction was performed using the Total RNA Purification Kit (Norgen Biotek) and RT-PCRs were carried out using the SuperScript™ III One-Step RT-PCR System with Platinum™ *Taq* DNA Polymerase (ThermoFisher Scientific) as per manufacturer’s indications. The following primers were used: SJ122: 5′-GGCATCACGGAAAATAGACG-3′ and PR296: 5′-CCTCCAGGGTGATGGTCTTG-3′ to detect construct-derived *dsx* and SJ90: 5′-CTGAATTACGCAGGCAGTGA-3′ and SJ89: 5′-ATGGTCGCATCGCTACAAGT-3′ to detect endogenous *Sfdsx.*

### Penetrance of the conditional male-selecting self-limiting trait and homozygosis

The penetrance of the conditional, male-selecting, self-limiting trait was assessed for each strain by rearing cohorts of first-instar larvae to adulthood on either on- or off-doxycycline diet. These larvae were the progeny of OX5382-hemizygous moths crossed with wild-type counterparts. Assuming no differences in survival rates between strains and sexes, a 1:1:1:1 ratio of OX5382-hemizygous male:OX5382-hemizygous female:wild-type male:wild-type female larvae is expected, with significant deviation from this ratio indicating mortality related to a given phenotype: in the case of OX5382-transformed strains, we anticipate mortality of OX5382 female larvae in the absence of dietary doxycycline. Upon pupation, pupae were collected and counted. Pupae from each treatment group were screened for DsRed2 fluorescence, and males and females were separated. Pupae were placed into cages according to phenotype to allow for assessment of adult eclosion rates. Strains exhibiting high female-specific mortality and high female survival when reared without and with doxycycline in the larval feed, respectively, were used for the establishment of homozygous colonies.

The OX5382G genomic insertion site was determined by splinkerette PCR [60] and primers specific to the integration site were designed. To identify OX5382G-homozygotes, single legs taken from individual, anaesthetised moths were PCR-genotyped using primers specific for the genomic sequence flanking the OX5382G insertion and for the OX5382 construct [61]. Primer sequences are available upon request.

### Mating competitiveness of the OX5382G strain

The mating competitiveness of the homozygous OX5382G males reared in the absence of doxycycline was assessed by measuring male pupal size and the proportion of paternity obtained by OX5382G males relative to wild-type males. Prior to eclosion, 100 OX5382G and 100 wild-type male pupae were weighed to examine whether average pupal weight differs between the transgenic and wild-type strains. Pupal weight data were analyzed using a general linear model. Cages were set up (30 × 30 × 30 cm) containing pupae from each of the three phenotypes and monitored for eclosion daily. Wild-type females were maintained in the eclosion cages, whereas male moths from both strains were transferred to holding containers each day to pair cohorts of wild-type males and OX5382G males of equivalent adult age per replicate.

To set up each cage (30 × 30 × 30 cm), 16 wild type males and 16 OX5382G males of comparable adult ages were introduced. One hour later, 15 wild type females of mixed adult age were then introduced to the cage. In total, 13 replicate cages were prepared. After 24 h, females were carefully removed from cages and placed in 4 oz. plastic pots, along with sugar water and filter paper for oviposition. Pots containing females were monitored daily for oviposition; if any eggs were present, they were transferred to a labelled Petri dish along with a small cube of on-dox diet and incubated at 26 °C [± 2 °C], > 75% relative humidity. Egg rafts were monitored daily for hatching. Upon hatching, 100 first-instar larvae per raft were screened for the presence or absence of the DsRed2 fluorescent marker. If fluorescent larvae were present, the female was considered to have mated with an OX5382G male, whilst if larvae did not exhibit fluorescence the female was considered to have mated with a wild-type male; no egg rafts giving rise to both fluorescent and non-fluorescent larvae were observed. To test for difference in mating success, the proportion of wild-type and fluorescent progeny were fitted to a generalized linear model with a binomial distribution. All analyses were performed using R version 4.0.0. [62].

### Post-release decline of transgene allele frequency

We conducted a laboratory study of caged populations of moths to estimate the rate at which the male-selecting self-limiting OX5382G trait would decline and fall to extinction in a population. All strains were reared as described above. OX5382G-hemizygous eggs were obtained by outcrossing OX5382G-homozygous males to wild-type females, and their eggs were collected. OX5382G-hemizygous eggs were reared in the absence of doxycycline, and pupae were sexed and screened for fluorescence. Thirty OX5382G-hemizygous males were crossed to 40 wild-type females, and eggs were collected. This first generation represents a post-release field population with a male-selecting trait frequency of approximately 0.25. Single larvae from three cohorts of 224 first-instar larvae from the first generation were each placed into individual diet cells with doxycycline-free diet. Each of the three cohorts represent a distinct population, and populations were closed and isolated from each other for the remainder of the experiment. Upon pupation, F_1_ pupae from each population were screened for fluorescence, sex-sorted, and counted. Males (with both OX5382G and wild type together) and females were kept in separate cages to allow eclosion and sexual maturation. Three to five days after the first male eclosed, females were transferred to the male cage for mating. At the peak of oviposition, ovipositional papers were collected, and eggs were washed to break up egg rafts and mix the progeny. Approximately 1000 eggs were randomly selected and left to hatch. Upon hatching, 224 first-instar larvae were randomly selected to establish the next generation and were placed individually into diet cells. This process was repeated until the OX5382G allele was eliminated from each population, as indicated by the absence of DsRed2-fluorescent individuals in the population.

### Modelling *Bt* trait resistance dilution

To model the effect of releasing OX5382G moths on resistance allele frequency and population size, a simulation-based deterministic population genetics model was created. The structure of the model was based on that described by Alphey et al. [33], with the addition of a second trait that confers resistance to a second protein. The model was created using the programming language R, version 4.0.0 [63].

The model assumes that: generations are discrete and do not overlap; there is one release of OX5382G insects that occurs once per generation; the initial population size is at carrying capacity; fitness penalties imposed by the self-limiting gene occurs during the larval stage; there is no fitness penalty associated with *Bt* resistance alleles; mating is random; the population experiences no immigration or emigration; larvae do not move from the plant on which they were laid as eggs; the self-limiting gene is not genetically linked to any resistance genes; and eggs have an equal chance of being male or female.

The model starts by creating a matrix that contains all possible genotypic combinations: for instance, if there are 2 *Bt* traits and one self-limiting gene, the number of possible genotypes is 3^3^ = 27 genotypes. Genotype and gamete frequencies will be different for males and females because of the female-specific lethality of the OX5382G gene, so male and female genotype frequencies are tracked separately. The model then assigns initial genotype frequencies by multiplying the allele frequencies that were inputted into the model. The model also assigns relative fitness associated with each of those genotypes ($\Omega_{i}$) using the following equation, where $\omega$ is the relative fitness of insects with genotype $i$ on transgenic plants, $\nu$ is the relative fitness of insects on refuge plants, and $\Phi$ is the proportion of refuge plants in the field.:

$$\Omega_{i} = \omega_{i} \Phi + \nu_{i} \left( {1 - \Phi } \right)$$

In models with two insecticidal (or *Bt*) proteins, $\omega$ is the product of the relative fitness of insects on plants with ‘protein 1’ and plants with ‘protein 2’, and $\nu$ is the product of relative fitnesses of insects on refuge plants.

The model then continues to go through the following steps:

1. Simulates releasing OX5382G males into the target population (which are assumed to all be adults) by changing genotype frequencies. The genotype frequency of released OX5382G males becomes $d/\left( {d + 1} \right)$, where $d$ is the over-flooding rate. All other genotype frequencies are multiplied by $1 - \left( {d/\left( {d + 1} \right)} \right)$ to make the sum of all genotype frequencies total 1.
2. Calculates the genotype frequencies of all possible gametes the adult population can produce.
3. Calculates the genotype frequencies of all zygotes that can be produced by the union of all combinations of sperm and egg.
4. Calculates the relative proportion of zygotes of each genotype surviving to adulthood by multiplying the zygote genotype frequency by the relative fitness associated with that genotype. Stores the number of insects that reach adulthood relative to the previous generation, then recalculates genotype frequencies to sum to 1.
5. Counts and stores the frequency of resistance alleles.
6. Calculates the change in population size (see details below).
7. The genotype frequencies of the current generation replace those of the previous generation, and the model returns to step 1, for a total of 100 simulated generations.

The relative population size in the next generation was calculated differently if the population was increasing or decreasing, since logistic equations are appropriate to calculate population growth, but not population decline [64]. If population size was decreasing, we calculated population size in the next generation ($N_{t + 1}$) using an exponential equation:

$$N_{t + 1} = N_{t} RF_{t} \sigma_{t}$$

where $N_{t}$ is the population size of the current generation, $R$ is the number of offspring a female can produce that live to reproductive age, $F_{t}$ is the proportion of the population in the current generation that is female, and $\sigma_{t}$ is the proportion of insects that survive to adulthood. An artificial lower limit to population size was built in, at 1% of carrying capacity to avoid creating infinitesimally small population sizes and to present a more conservative and realistic simulation. If population size was increasing, population size in the next generation was calculated using a logistic equation:

$$N_{t + 1} = \frac{{N_{t} RF_{t} \sigma_{t} }}{{1 + N_{t} \left( {RF_{t} \sigma_{t} - 1} \right)}}$$

Note that population size calculations do not directly include resistance allele frequencies, and conversely, allele frequency calculations are made independent of population size.

In the modelled scenarios, ‘Protein 1’ represents a new insecticidal protein produced by biotech corn. Genetic resistance to the protein occurs in the moth population, but at a very small frequency (0.005). The probability of survival of susceptible insects on corn expressing this protein is low (0.01), as is the survival of insects only carrying one resistance allele (0.02). ‘Protein 2’ represents an older protein produced by biotech corn, to which insects have had longer exposure, and have higher starting resistance frequencies (0.1), and higher survival of homozygous susceptible (0.2) and heterozygous (0.4) genotypes. It is possible that resistance alleles occur in the laboratory population at a very low frequency, so this value was assumed to be the same as the resistance frequency present in the naive wild-type population (0.005) (however, if OX5382G moths were to be released for its intended use, the line would be tested to ensure a resistance allele frequency of less than 0.001) (Table 1).

**Table 1:** Starting parameters of the modelled scenarios

Parameter	Protein 1	Protein 2
Relative fitness of ss genotype on transgenic plant (*Ω*_*ss*_)	0.01	0.2
Relative fitness of rs genotype on transgenic plant (*Ω*_*rs*_)	0.02	0.4
Relative fitness of rr genotype on transgenic plant (*Ω*_*rr*_)	1	1
Relative fitness of all genotypes on refuge plants	1	1
Initial resistance allele frequency (*p*_*r*_)	0.005	0.1
Frequency of resistance alleles in released OX5382G	0.005	
Number of insecticidal proteins produced by the crop	1 or 2	
Proportion of the crop that is refuge	1%, 5%, or 10%	
Number of offspring per female that survive to adulthood ($R$)	5	

ss, homozygous susceptible genotype; rs, heterozygous genotype; rr, homozygous resistant genotype

The difference in over-flooding rates (*d*) necessary to cause population suppression and lower resistance allele frequencies was observed in modelled scenarios:

1. When plants express one versus two *Bt* proteins. In this scenario, the amount of refuge is constant at 10%.
2. When the percentage of refuge plants is lowered from 10% to 5% to 1%. In this scenario, the number of insecticidal proteins is constant at 2.

## Supplementary Information

**Additional file 1: Figure S1:** Alignment of OX5382 *dsx* and endogenous *Sfdsx* coding sequence. Differences between the two coding sequences, introduced to generate a female-specific open reading frame, are highlighted. Different exons are boxed in grey and red. The ‘TAG’ stop codon in-frame with the male-specific splice variant of OX5382 *dsx* is underlined.

## Data Availability

The datasets used and/or analysed during the current study are available from the corresponding author on reasonable request.

## Abbreviations

*Bt*: *Bacillus thuringiensis*
*Ac*MNPV: *Autographa californica* Multicapsid nuclear polyhedrosis virus
*DmHsp70*: *Drosophila melanogaster* Heat shock protein 70
*dsx*: *doublesex*
EST: Expressed sequence tag
G_0_, G_1_: Generation 0, generation 1
*Sfdsx*: *Spodoptera frugiperda doublesex*
*tetO*_7_: Tetracycline-responsive operator sequence
tTAV: Tetracycline-repressible transactivator protein variant
