# Supplementary figures and images for: Self-limiting fall armyworm: a new approach in development for sustainable crop protection and resistance management
Source: BMC Biotechnol. 2022 Jan 27;22:5. doi: 10.1186/s12896-022-00735-9 (PMC8793274; doi:10.1186/s12896-022-00735-9)

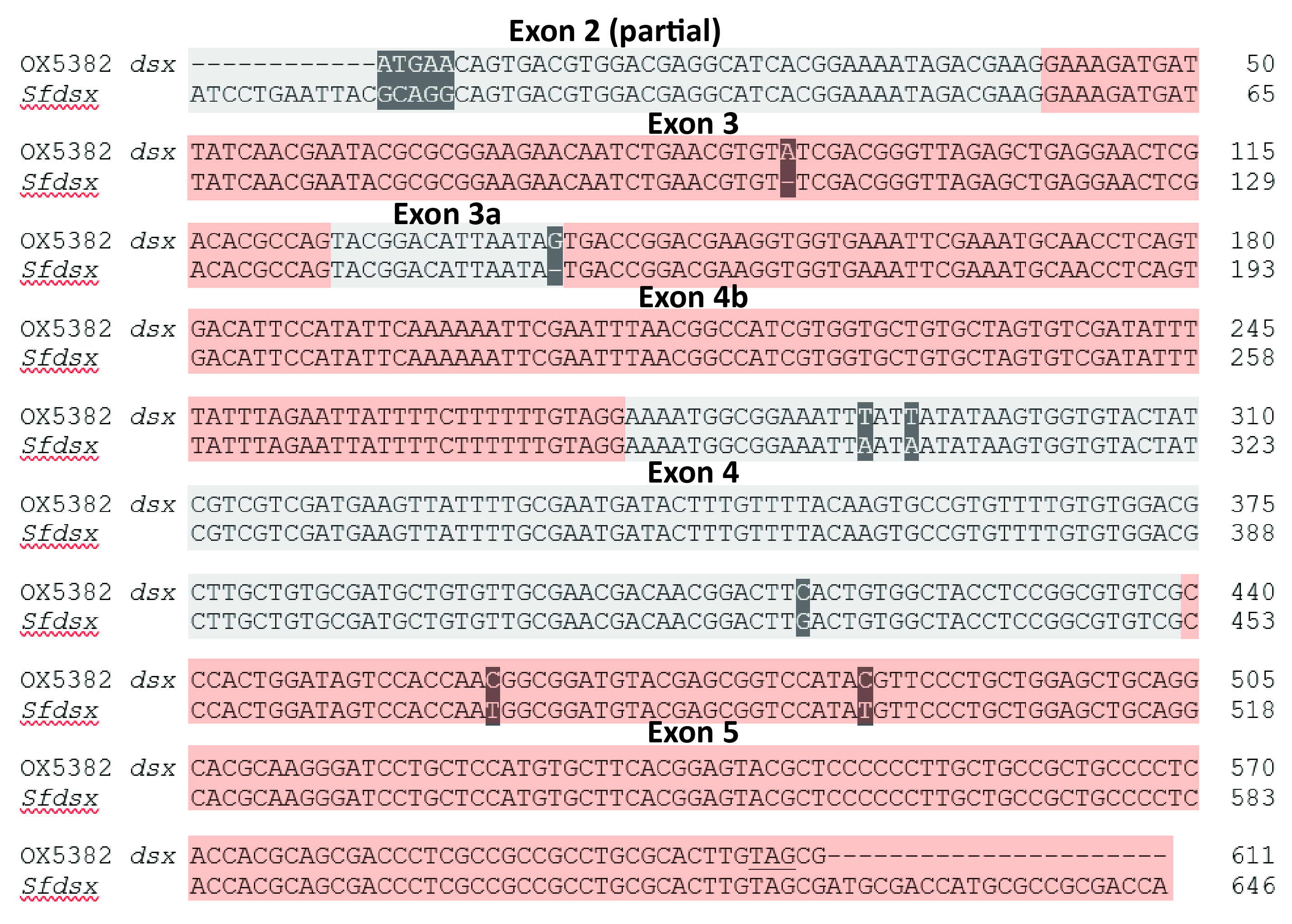

Supplement: Supplementary file 1 — Additional file 1: Figure S1: Alignment of OX5382 dsx and endogenous Sfdsx coding sequence. Differences between the two coding sequences, introduced to generate a female-specific open reading frame, are highlighted. Different exons are boxed in grey and red. The ‘TAG’ stop codon in-frame with the male-specific splice variant of OX5382 dsx is underlined. [file 12896_2022_735_MOESM1_ESM.jpg]
